# Prognostic value of pretreatment Controlling Nutritional Status score in esophageal cancer: a meta-analysis

**DOI:** 10.3389/pore.2023.1611221

**Published:** 2023-06-27

**Authors:** Jing Lv, Peirui Chen, Jianqiang Wu, Caihong Hu

**Affiliations:** Department of Thoracic Surgery, People’s Hospital of Deyang City, Deyang, China

**Keywords:** meta-analysis, prognosis, esophageal cancer, pretreatment, Controlling Nutritional Status score

## Abstract

**Background and purpose:** The association between the pretreatment Controlling Nutritional Status (CONUT) score and the prognosis of esophageal cancer patients remains unclear. The aim of this meta-analysis was to further elucidate the prognostic role of the pretreatment CONUT score in esophageal cancer based on current evidence.

**Methods:** The PubMed, Embase, Web of Science and CNKI databases were searched up to 27 September 2022. The primary and secondary outcomes were overall survival (OS) and progression-free survival (PFS)/cancer-specific survival (CSS), and the hazard ratio (HR) and 95% confidence interval (CI) were pooled for analysis.

**Results:** A total of 11 retrospective studies involving 3,783 participants were included. The pooled results demonstrated that a higher pretreatment CONUT score was significantly related to poor OS (HR = 1.82, 95% CI: 1.31–2.54, *p* < 0.001), and subgroup analysis stratified by pathological type showed similar results. In addition, the pretreatment CONUT score was associated with poor PFS (HR = 1.19, 95% CI: 1.10–1.28, *p* < 0.001) and CSS (HR = 2.67, 95% CI: 1.77–4.02, *p* < 0.001).

**Conclusion:** The pretreatment CONUT score was predictive of worse prognosis in esophageal cancer, and patients with a higher CONUT score showed worse survival.

## Introduction

Esophageal carcinoma is one of the most common malignant cancers worldwide, ranking sixth and seventh in terms of mortality and incidence [1, 2]. To date, the prognosis of patients with esophageal cancer remains extremely poor despite considerable advances in surgical technologies, neoadjuvant chemotherapy and immunotherapy [3]. The TNM stage is regarded as the prognostic indicator for esophageal cancer and contributes substantially to the formulation of a treatment strategy. However, increasing evidence indicates that a number of host parameters affect the long-term survival of esophageal cancer [4].

In particular, nutritional status has been verified as an essential prognostic factor in cancers, and it has been reported that impaired nutritional conditions have a negative impact on survival in cancer patients [5]. Thus, a number of nutritional indicators based on laboratory data have been investigated, and the prognostic role of several indices has been explored in recent years, including the prognostic nutritional index (PNI) [6], nutritional risk index (NRI) [7], geriatric nutritional risk index (GNRI) [8] and modified Glasgow Prognostic Score (mGPS) [9]. Some of them have been shown to be significantly associated with the prognosis of esophageal carcinoma patients [6, 8, 9]. The Controlling Nutritional Status (CONUT) score is a novel nutritional indicator that has been described in the past several years [10]. It is calculated by scoring serum, albumin concentration, lymphocyte count and total cholesterol level [11], and the association of CONUT score and perioperative surgical risk in esophageal cancer has been reported [12]. In addition, the relationship between the pretreatment CONUT score and survival has also been manifested in several types of tumors by meta-analyses, including renal cell carcinoma, pancreatic cancer, lung cancer and colorectal cancer [13–16]. However, whether the pretreatment CONUT score could serve as a reliable prognostic factor in esophageal cancer is unclear.

Therefore, this meta-analysis aimed to further elucidate the association of the pretreatment CONUT score with long-term survival in esophageal cancer, which might contribute to the treatment strategy for esophageal cancer patients.

## Materials and methods

This meta-analysis was performed according to the Preferred Reporting Items for Systematic Review and Meta-Analyses 2020 [17].

### Literature search

The PubMed, Embase, Web of Science and CNKI databases were searched up to 27 September 2022, for studies investigating the prognostic role of the pretreatment CONUT score in esophageal cancer. The following terms were used during the search: Controlling Nutritional Status score, CONUT, esophageal, esophagus, cancer, tumor, carcinoma, neoplasm, survival, prognostic and prognosis. The specific search strategy was as follows: (Controlling Nutritional Status score OR CONUT) AND (esophageal OR esophagus) AND (cancer OR tumor OR carcinoma OR neoplasm) AND (survival OR prognostic OR prognosis). Additionally, all references cited in the included studies were also screened.

### Inclusion criteria

The inclusion criteria were as follows: 1) patients were diagnosed with primary esophageal cancer pathologically; 2) the CONUT score was calculated based on the serum albumin concentration, peripheral total lymphocyte count and cholesterol level before antitumor treatment as previously described: serum albumin levels≥3.50 g/dL, 3.00–3.49 g/dL, 2.50–3.49 g/dL and <2.50 g/dL [18] were separately defined as score 0, 2, 4 and 6, the total cholesterol levels≥180 mg/dL, 140–179 mg/dL, 100–139 mg/dL and<100 mg/dL were scored 0, 1, 2 and 3 and the total lymphocyte counts ≥1,600/µL, 1,200–1,599/µL, 800–1,199/µL and<800/µL were scored 0, 1, 2 and 3, respectively; 3) patients were divided into two groups based on the CONUT score and the association between pretreatment CONUT score and prognosis presenting as the overall survival (OS), progression-free survival (PFS) or cancer-specific survival (CSS); 4) the hazard ratios (HRs) and 95% confidence intervals (CIs) for the above endpoints were provided in the papers or the Kaplan‒Meier survival curves were presented; and 5) high-quality studies with a Newcastle‒Ottawa Scale (NOS) score >5 [19].

### Exclusion criteria

The exclusion criteria were as follows: 1) publications with letters, editorials, case reports, reviews or animal trials; 2) overlapped or duplicated data; and 3) the HRs with corresponding 95% CIs were not available.

### Data collection

The following information was extracted: the name of the first author, publication year, sample size, country, tumor-node-metastasis (TNM) stage, treatment (surgical or nonsurgical therapy), pathological type, definition and comparison of CONUT score, endpoint, HR and 95% CI.

### Methodological quality assessment

The quality was assessed according to the NOS score tool, and only high-quality studies with an NOS score ≥5 were ultimately included [19].

The literature search, selection, data collection and quality assessment were all conducted by two authors independently, and any disagreement was resolved by discussion.

### Statistical analysis

All statistical analyses were performed using STATA 15.0 software. The HRs and 95% CIs were calculated to evaluate the association between the pretreatment CONUT score and the prognosis of esophageal cancer patients. Heterogeneity among the included studies was evaluated using I^2^ statistics and the Q-test. If obvious heterogeneity was observed, representing I^2^ > 50% and/or *p* < 0.1, the random effects model was applied; otherwise, the fixed effects model was used. Sensitivity analysis was performed to clarify sources of heterogeneity and evaluate the stability of the pooled results. Additionally, Begg’s test and Egger’s test were conducted to detect publication bias, and significant publication bias was defined as *p* < 0.05 [20, 21]. If we detected significant publication bias, then the nonparametric trim-and-fill method was used to re-estimate a corrective effect size after publication bias was adjusted [22].

## Results

### Literature search and selection

**FIGURE 1:**
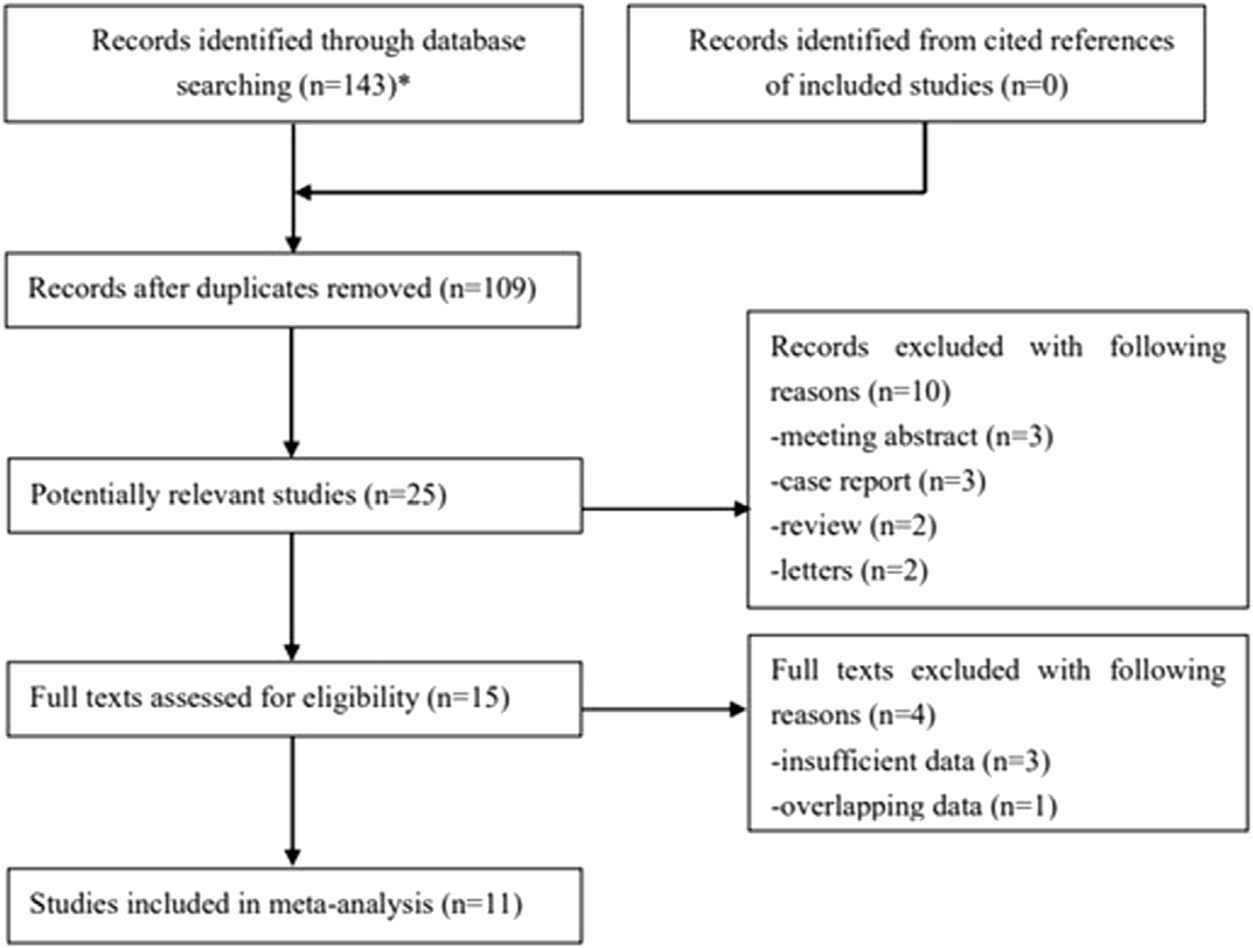
The flow diagram of this meta-analysis.

One hundred forty-three records were searched from databases, and 34 duplicated publications were removed. Then, 25 potentially relevant studies were further reviewed after excluding 84 irrelevant publications by reading the titles. The full texts of the remaining 15 studies were carefully reviewed after excluding 10 publications. Ultimately, 11 retrospective studies were included [23–33]. The specific process is displayed in Figure 1.

### Basic characteristics of the included studies

A total of 3,783 patients were enrolled, and most (7/11) studies were from Japan [23–25, 27–30]. Except for the study by Chang et al. [33], the other studies included operated patients. In addition, six studies focused on esophageal squamous cell carcinoma (ESCC) patients [23, 25, 26, 29, 31, 32]. The other detailed characteristics are presented in Table 1.

**TABLE 1:** Basic characteristics of included studies.

Author	Year	Sample size	Country	TNM stage	Treatment	Pathological type	Definition of CONUT score and comparison	Endpoint	NOS
Toyokawa [23]	2016	185	Japan	I-IV	Surgery	SCC	Albumin (g/dL): ≥3.50: 0, 3.00–3.49: 2, 2.50–2.99: 4, <2.50: 6; lymphocyte count (/mm^3^): ≥1,600: 0, 1,200–1,599: 1, 800–1,199: 2, <800: 3; cholesterol (mg/dL): ≥180: 0, 140–179: 1, 100–139: 2, <100: 3; CONUT 0–1 vs. ≥ 2	OS, PFS	7
Yoshida [24]	2017	373	Japan	I-IV	Surgery	EC	Albumin (g/dL): ≥3.50: 0, 3.00–3.49: 2, 2.50–2.99: 4, <2.50: 6; lymphocyte count (/mm^3^): ≥1,600: 0, 1,200–1,599: 1, 800–1,199: 2, <800: 3; cholesterol (mg/dL): ≥180: 0, 140–179: 1, 100–139: 2, <100: 3; NR	OS, CSS	7
Hirahara [25]	2018	148	Japan	I-III	Surgery	SCC	Albumin (g/dL): ≥3.50: 0, 3.00–3.49: 2, 2.50–2.99: 4, <2.50: 6; lymphocyte count (/mm^3^): ≥1,600: 0, 1,200–1,599: 1, 800–1,199: 2, <800: 3; cholesterol (mg/dL): ≥180: 0, 140–179: 1, 100–139: 2, <100: 3; CONUT 0–1 vs. 2–4 vs. 5–12	CSS	7
Xu [26]	2018	510	China	I-IV	Surgery	SCC	Albumin (g/dL): ≥3.50: 0, 3.00–3.49: 2, 2.50–2.99: 4, <2.50: 6; lymphocyte count (/mm^3^): ≥1,600: 0, 1,200–1,599: 1, 800–1,199: 2, <800: 3; cholesterol (mg/dL): ≥180: 0, 140–179: 1, 100–139: 2, <100: 3; CONUT 0–1 vs. ≥ 2	OS	6
Hikage [27]	2019	141	Japan	I-IV	Surgery	EC	Albumin (g/dL): ≥3.50: 0, 3.00–3.49: 2, 2.50–2.99: 4, <2.50: 6; lymphocyte count (/mm^3^): ≥1,600: 0, 1,200–1,599: 1, 800–1,199: 2, <800: 3; cholesterol (mg/dL): ≥180: 0, 140–179: 1, 100–139: 2, <100: 3; CONUT 0–4 vs. 5–12	OS, PFS	6
Sakai [28]	2020	105	Japan	I-IV	Surgery	EC	Albumin (g/dL): ≥3.50: 0, 3.00–3.49: 2, 2.50–2.99: 4, <2.50: 6; lymphocyte count (/mm^3^): ≥1,600: 0, 1,200–1,599: 1, 800–1,199: 2, <800: 3; cholesterol (mg/dL): ≥180: 0, 140–179: 1, 100–139: 2, <100: 3; CONUT <3 vs. ≥ 4	OS	6
Suzuki [29]	2021	241	Japan	I	Surgery	SCC	Albumin (g/dL): ≥3.50: 0, 3.00–3.49: 2, 2.50–2.99: 4, <2.50: 6; lymphocyte count (/mm^3^): ≥1,600: 0, 1,200–1,599: 1, 800–1,199: 2, <800: 3; cholesterol (mg/dL): ≥180: 0, 140–179: 1, 100–139: 2, <100: 3; CONUT 0–1 vs. ≥ 2	OS	6
Urabe [30]	2021	224	Japan	NR	Surgery	EC	Albumin (g/dL): ≥3.50: 0, 3.00–3.49: 2, 2.50–2.99: 4, <2.50: 6; lymphocyte count (/mm^3^): ≥1,600: 0, 1,200–1,599: 1, 800–1,199: 2, <800: 3; cholesterol (mg/dL): ≥180: 0, 140–179: 1, 100–139: 2, <100: 3; CONUT 0–1 vs. 2–4 vs. 5–12	OS, CSS	8
Xu [31]	2021	522	China	I-IV	Surgery	SCC	Albumin (g/dL): ≥3.50: 0, 3.00–3.49: 2, 2.50–2.99: 4, <2.50: 6; lymphocyte count (/mm^3^): ≥1,600: 0, 1,200–1,599: 1, 800–1,199: 2, <800: 3; cholesterol (mg/dL): ≥180: 0, 140–179: 1, 100–139: 2, <100: 3; CONUT 0–1 vs. ≥ 2	OS	6
Yoon [32]	2021	1265	Korea	I-IV	Surgery	SCC	Albumin (g/dL): ≥3.50: 0, 3.00–3.49: 2, 2.50–2.99: 4, <2.50: 6; lymphocyte count (/mm^3^): ≥1,600: 0, 1,200–1,599: 1, 800–1,199: 2, <800: 3; cholesterol (mg/dL): ≥180: 0, 140–179: 1, 100–139: 2, <100: 3; CONUT 0–2 vs. ≥ 3	OS, PFS	7
Chang [33]	2022	69	China	Advanced	Non-surgery	EC	Albumin (g/dL): ≥3.50: 0, 3.00–3.49: 2, 2.50–2.99: 4, <2.50: 6; lymphocyte count (/mm^3^): ≥1,600: 0, 1,200–1,599: 1, 800–1,199: 2, <800: 3; cholesterol (mg/dL): ≥180: 0, 140–179: 1, 100–139: 2, <100: 3; CONUT 0–1 vs. ≥ 2	OS, PFS	7

NR, not reported; SCC, squamous cell carcinoma; EC, esophageal cancer; CONUT, controlling nutritional status; OS, overall survival; PFS, progression-free survival; CSS, cancer-specific survival; NOS, Newcastle-Ottawa Scale.

### Predictive effect of the pretreatment CONUT score for OS

**FIGURE 2:**
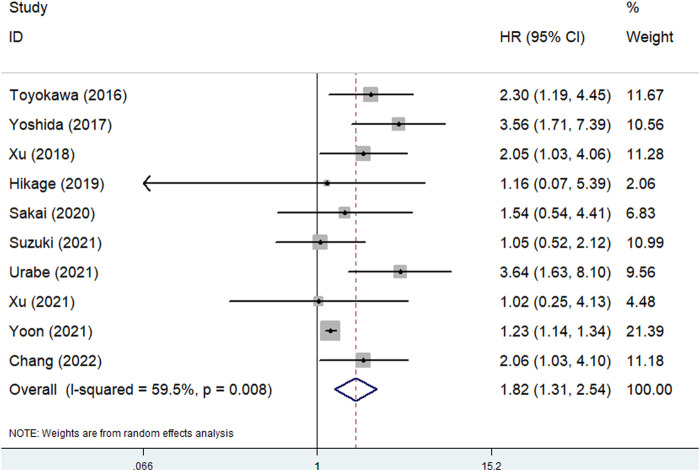
The association between pretreatment CONUT score and overall survival of esophageal cancer patients.

Ten studies explored the predictive effect of the pretreatment CONUT score on OS in esophageal cancer [23, 24, 26–33]. The results demonstrated that a higher pretreatment CONUT score was obviously related to poorer OS (HR = 1.82, 95% CI: 1.31–2.54, *p* < 0.001; I^2^ = 59.5%, *p* = 0.008) (Figure 2). Subgroup analysis based on the pathological type showed similar results (ESCC: HR = 1.40, 95% CI: 1.06–1.85, *p* = 0.019; esophageal cancer: HR = 2.59, 95% CI: 1.76–3.82, *p* < 0.001) (Table 2).

**TABLE 2:** Results of included studies.

	No. of studies	Hazard ratio	95% confidence interval	*p*-value	I^2^ (%)	*p*-value
Overall survival	10 [23, 24, 26–33]	1.82	1.31–2.54	<0.001	59.5	0.008
Pathological type						
Squamous cell carcinoma	5 [23, 26, 29, 31, 32]	1.40	1.06–1.85	0.019	30.7	0.217
Esophageal cancer	5 [24, 27, 28, 30, 33]	2.59	1.76–3.82	<0.001	0.0	0.509
Progression-free survival	4 [23, 27, 32, 33]	1.19	1.10–1.28	<0.001	19.9	0.290
Cancer-specific survival	3 [24, 25, 30]	2.67	1.77–4.02	<0.001	0.0	0.494

### Predictive effect of pretreatment CONUT score on PFS and CSS

**FIGURE 3:**
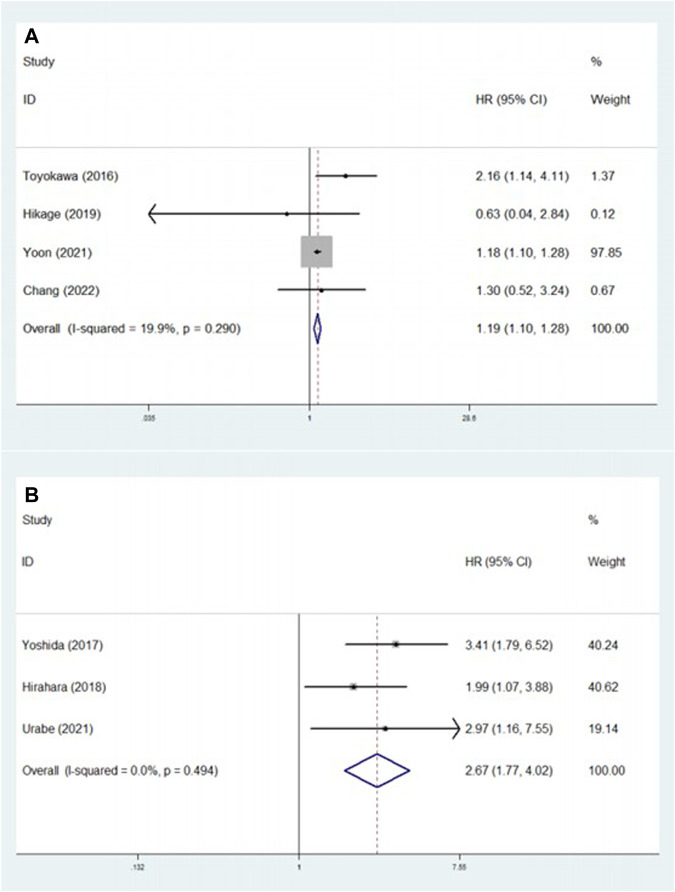
**(A)** The association between pretreatment CONUT score and progression-free survival of esophageal cancer patients. **(B)** The association between pretreatment CONUT score and cancer-specific survival of esophageal cancer patients.

Four [23, 27, 32, 33] and three [24, 25, 30] studies explored the association between pretreatment CONUT score and PFS and CSS, respectively. The pooled results indicated that a higher pretreatment CONUT score predicted worse PFS (HR = 1.19, 95% CI: 1.10–1.28, *p* < 0.001; I^2^ = 19.9%, *p* = 0.290) and CSS (HR = 2.67, 95% CI: 1.77–4.02, *p* < 0.001; I^2^ = 0.0%, *p* = 0.494) (Figures 3A, B; Table 2).

### Sensitivity analysis and publication bias

**FIGURE 4:**
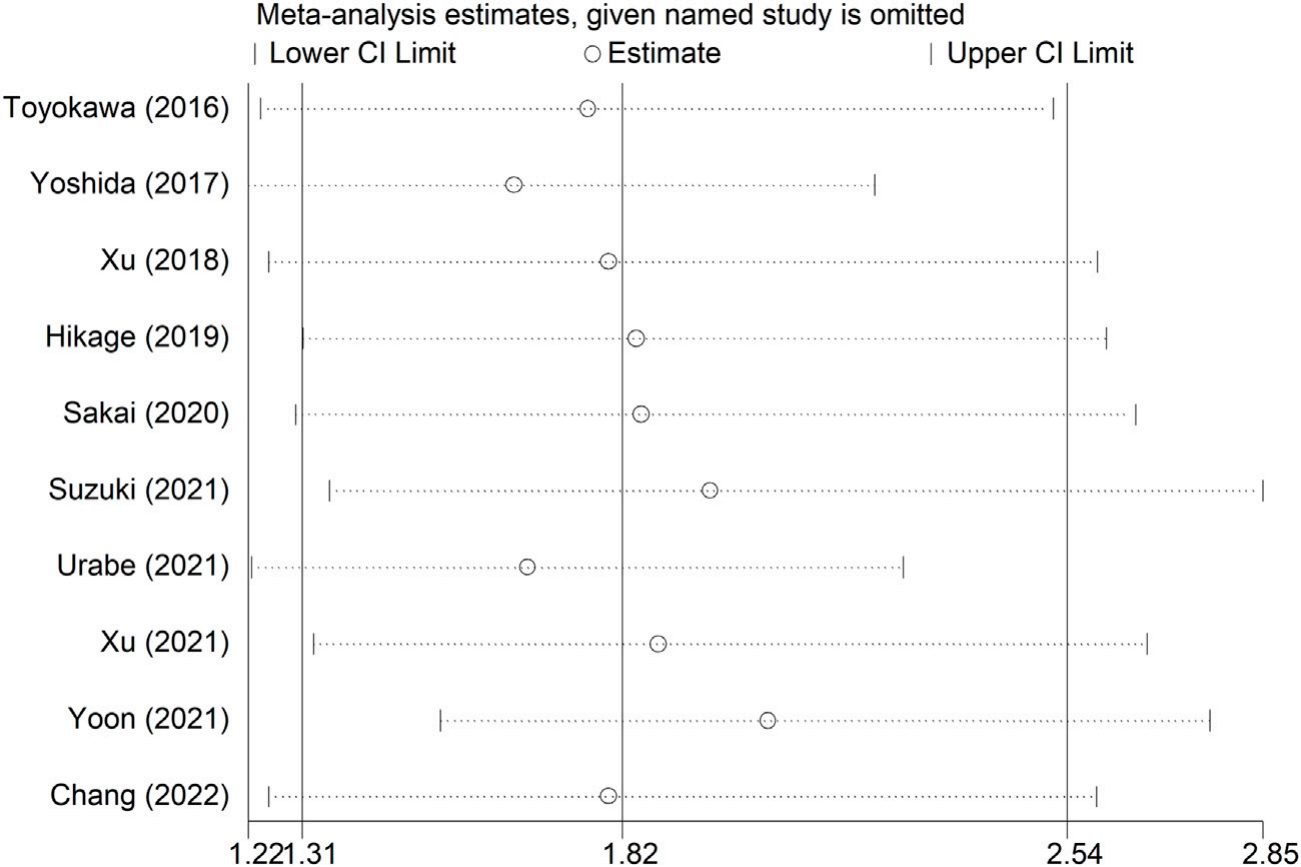
Sensitivity analysis for the overall survival.

**FIGURE 5:**
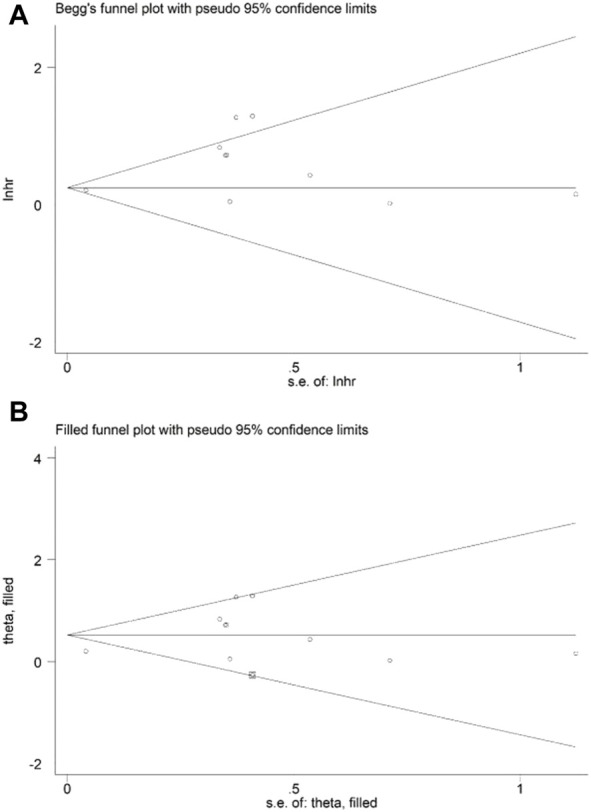
Begg’s **(A)** and filled Begg’s **(B)** funnel plots.

Sensitivity analysis for OS showed that the results of the current meta-analysis were reliable and stable, and none of the included studies showed an obvious impact on the overall results (Figure 4). However, based on the asymmetric Begg’s funnel plot (Figure 5A) and *p* = 0.050 of Egger’s test, significant publication bias was revealed. Thus, the nonparametric trim-and-fill method was conducted, and only one potentially “unpublished” study was revealed (Figure 5B), but this study did not affect the overall results (filled HR = 1.69, 95% CI: 1.24–2.30, *p* = 0.001).

## Discussion

This meta-analysis showed that the pretreatment CONUT score was associated with the prognosis of esophageal cancer patients, and patients with a higher pretreatment CONUT score experienced poorer OS, PFS and CSS based on current evidence. Therefore, the pretreatment CONUT might contribute to the evaluation of long-term survival and formulation of therapy strategies for esophageal cancer patients. However, because of limitations in this meta-analysis, more high-quality prospective studies are needed to verify the above findings.

The CONUT score consisted of serum albumin concentration, total cholesterol level and total lymphocyte count in peripheral blood. Serum albumin usually reflects the ability of the body to synthesize protein, total cholesterol level reflects the ability of the body to metabolize lipids, and peripheral total lymphocyte count mainly reflects the immune condition of the body [23]. Previous studies have well demonstrated that the nutritional and immune-inflammatory status is closely associated with the development, progression and prognosis of cancers. As mentioned above, the serum and cholesterol and lymphocyte count are good indicators for nutritional and immune-inflammatory status of patients, respectively. Therefore, CONUT score is indicated to be closely related to prognosis of esophageal cancer patients. Overall, the CONUT score is an easier and more objective assessment of nutritional status than the Subjective Global Assessment and the Full Nutritional Assessment [34]. In addition, we deem that the CONUT score might have some advantages over other nutritional indicators, such as the PNI, NRI and GNRI, mentioned above. PNI was calculated as follows: PNI = 10 × serum albumin (g/dL) + 0.005 × total lymphocyte count (per mm^3^) [6], NRI was calculated according to the following formula: NRI = 1.519 × albumin (g/dL) + 41.7 × present weight/usual weight [35], and GNRI was calculated as follows: GNRI = 1.489 × albumin (g/dL) + 41.7 × present weight/ideal weight [36]. First, cholesterol plays an essential role in the progression and prognosis of esophageal cancer patients. It has been verified that the cholesterol level in tumor tissues is higher than that in normal tissues and that the cholesterol metabolite 27-hydroxycholesterol promotes the proliferation and migration of tumor cells [37, 38]. Tao et al. demonstrated that Lysophosphatidylcholine acyltransferase 1 (LPCAT1) reprograms tumor cells cholesterol metabolism in esophageal cancer and could be applied as a potential treatment target against esophageal carcinoma [39]. Furthermore, total cholesterol level was reported as a prognostic factor in esophageal cancer patients [40]. Thus, the cholesterol level in the peripheral blood, reflecting the condition of cholesterol metabolism to some extent, contributes to the evaluation of tumor progression and prognosis. In addition, the effect of body weight on the prognosis of cancer patients is polarizing. In detail, both overweight and low body weight are significantly associated with poor survival in cancer patients [41, 42]. Furthermore, the muscle content could objectively reflect the nutritional status of the body, and several indices, such as the skeletal muscle mass index (SMI), are reported to have high prognostic value in esophageal cancer [43, 44]. However, they are calculated from CT images in a relatively complicated way, which limits their application in clinics.

The prognostic role of the pretreatment CONUT score has been verified in several types of cancers. Ma et al. included seven studies with 2,294 patients and showed that an elevated CONUT score was related to poor OS (HR = 1.56, *p* = 0.007) in pancreatic cancer [14]. In addition, Takagi et al. demonstrated that a high CONUT score was related to poor OS (HR = 1.97, *p* < 0.001), CSS (HR = 3.64, *p* < 0.001) and PFS (HR = 1.68, *p* = 0.001) after reviewing 2,601 participants [13]. Furthermore, the association between the CONUT score and the prognosis of lung cancer patients was also identified in a meta-analysis by Zhang et al. [15]. Our meta-analysis further verified the predictive role of the CONUT score for the long-term survival of esophageal cancer patients.

There are still several fields about the prognostic value of the CONUT score in esophageal cancer that need further investigation. First, only the relationship between pretreatment CONUT score and survival was identified in most relevant studies. The posttreatment CONUT score and changes in the CONUT score during antitumor treatment might also contribute to the prediction of the therapeutic effect and long-term survival of esophageal cancer. In addition, it is necessary to identify the clinical value of moderately lowering the CONUT score before or during antitumor treatment. For example, whether reducing CONUT scores by consuming cholesterol-high foods and supplementing with albumin could improve the therapeutic effect to antitumor therapy remains unclear. Furthermore, it is also worth investigating whether increasing lymphocyte count before and during the anti-tumor treatment can improve patient outcomes.

There are several limitations in this meta-analysis. First, all included studies were retrospective, and the overall sample size was relatively small. Thus, some bias might exist and should be considered. Second, all patients were from Asian countries, which might limit the generalizability of our conclusions. Third, due to the lack of original data, we were unable to conduct subgroup analysis based on other important parameters, such as TNM stage and age.

## Conclusion

The pretreatment CONUT score was predictive of worse prognosis in esophageal cancer, and patients with a higher CONUT score showed worse survival. However, more high-quality prospective studies are still needed to verify our findings.

## Data Availability

The original contributions presented in the study are included in the article/supplementary material, further inquiries can be directed to the corresponding author.
